# Implicit structured sequence learning: an fMRI study of the structural mere-exposure effect

**DOI:** 10.3389/fpsyg.2014.00041

**Published:** 2014-02-04

**Authors:** Vasiliki Folia, Karl Magnus Petersson

**Affiliations:** ^1^Neurobiology of Language, Max Planck Institute for PsycholinguisticsNijmegen, Netherlands; ^2^Neurocognition of Language, Donders Institute for Brain, Cognition and Behaviour, Radboud University NijmegenNijmegen, Netherlands; ^3^Cognitive Neuroscience Research Group, Department of Psychology, Institute of Biotechnology and Bioengineering, Centre for Molecular and Structural Biomedicine (CBME), Universidade do AlgarveFaro, Portugal

**Keywords:** fMRI, artificial syntax, implicit learning, artificial grammar learning, inferior frontal gyrus, structural mere-exposure, preference classification

## Abstract

In this event-related fMRI study we investigated the effect of 5 days of implicit acquisition on preference classification by means of an artificial grammar learning (AGL) paradigm based on the structural mere-exposure effect and preference classification using a simple right-linear unification grammar. This allowed us to investigate implicit AGL in a proper learning design by including baseline measurements prior to grammar exposure. After 5 days of implicit acquisition, the fMRI results showed activations in a network of brain regions including the inferior frontal (centered on BA 44/45) and the medial prefrontal regions (centered on BA 8/32). Importantly, and central to this study, the inclusion of a naive preference fMRI baseline measurement allowed us to conclude that these fMRI findings were the intrinsic outcomes of the learning process itself and not a reflection of a preexisting functionality recruited during classification, independent of acquisition. Support for the implicit nature of the knowledge utilized during preference classification on day 5 come from the fact that the basal ganglia, associated with implicit procedural learning, were activated during classification, while the medial temporal lobe system, associated with explicit declarative memory, was consistently deactivated. Thus, preference classification in combination with structural mere-exposure can be used to investigate structural sequence processing (syntax) in unsupervised AGL paradigms with proper learning designs.

## Introduction

Artificial grammar learning (AGL) is commonly used to probe implicit sequence learning (Reber, 1967; Seger, 1994; Stadler and Frensch, 1998). In the standard AGL paradigm, participants are exposed to example sequences that are generated from a finite set of rules, a grammar, which specify non-overt (non-marked) sequence regularities. After exposure, participants classify new sequences as grammatical or not (*grammaticality instruction*). Participants that perform robustly above chance are said to have acquired relevant knowledge related to the grammar and their classification performance shows that they are able to generalize and use the acquired knowledge effectively in a new situation. Although AGL is often used to probe incidental implicit learning, most functional neuroimaging (and some recent behavioral) research has used explicit instructions in combination with the grammaticality classification task. In these experiments, the participants are informed about the existence of a grammar before acquisition (i.e., before exposure to grammatical items) and are explicitly instructed to identify the underlying rules by, for example, trial-and-error or other explicit problem solving strategies, in combination with performance feedback during acquisition and sometimes during classification. Participants in these studies are therefore explicitly guided in their learning toward what is relevant to learn and what is not (e.g., Fletcher et al., 1999; Strange et al., 2001; Opitz and Friederici, 2003; reviewed in Petersson et al., 2004; and more recently, Bahlmann et al., 2008, 2009; reviewed in Petersson et al., 2010). For example, in the studies by Opitz and Friederici (2003, 2007); Opitz and Kotz (2012), the participants were instructed to extract the underlying rules during training, while feedback was provided on each trial during testing. In contrast, implicit AGL studies avoid using explicit instructions for the acquisition session(s) and do not providing any sort of performance feedback, although the grammaticality instruction presupposes (at the time of classification) that the participants are informed about the existence of an underlying grammar.

A central aspect of implicit learning, the mere-exposure effect (Zajonc, 1968; see also Reber, 1967), is the observation that participants that have been exposed to stimuli show an enhanced preference for these compared to novel stimuli. The mere-exposure effect has been investigated with positron emission tomography and abstract visual stimuli (Japanese ideograms) and resulted in a right inferior frontal activation including Brodmann's area (BA) 44 (Elliott and Dolan, 1998). In contrast to this surface-based mere-exposure effect, the *structural* mere-exposure effect is based on an underlying rule-system for stimulus generation and is characterized by the tendency to prefer new stimuli that conform to the rule-system, independent of surface structure (Gordon and Holyoak, 1983; Zizak and Reber, 2004). In implicit AGL paradigms, the structural mere-exposure effect provides a sensitive indirect measure of grammatical knowledge (Zizak and Reber, 2004). *Preference classification*, in combination with a structural mere-exposure design, can therefore be used to investigate syntactic (structural) processing in unsupervised AGL paradigms. One difference between this type of paradigm and explicit AGL paradigms is that in the former, both the acquisition and classification phases are implicit and there is no reference to any previous acquisition episode made (Shanks and St. John, 1994). Because of this, it is never necessary to inform the participants about the existence of a generative grammar or any other aspect of the paradigm and the preference classification instruction minimizes the potential that participants develop and/or use deliberate explicit strategies (e.g., problem solving). In addition, from the subject's point of view there is no correct or incorrect response and the motivation to use explicit strategies is therefore further minimized during the experiment. More importantly from the point of view of functional neuroimaging is the fact that this paradigm allows us to acquire a naive classification baseline, both in terms of a proper behavioral preference classification baseline and a corresponding fMRI baseline in within-subject designs. This paradigm has been investigated behaviorally and we have shown in several experiments that participants classify robustly well-above chance on regular as well as non-regular grammars (Folia et al., 2008; Forkstam et al., 2008; Uddén et al., 2012). However, the learning paradigm has not been investigated with functional neuroimaging methods. Previous fMRI studies of implicit AGL (structural mere-exposure and grammaticality instruction) have shown that the grammaticality effect engages inferior frontal regions, centered on BA 44 and 45, and medial prefrontal region, centered on BA 8 and 32, as well as the basal ganglia (Petersson et al., 2004, 2010; Forkstam et al., 2006), while the medial temporal lobe memory system is deactivated (Petersson et al., 2010). This raises the question whether these findings reflect intrinsic outcomes of the learning process itself or whether they reflect a preexisting functionality that is recruited during classification. In this study, we address this issue by investigating the neural correlates of incidental structured sequence learning by means of a multi-day implicit AGL paradigm based on preference classification in a structural mere-exposure design. On the first day, before the first acquisition session, we acquired event-related fMRI data in order to establish a naive preference baseline by asking the participants to indicate whether they liked or disliked sequences based to their immediate intuitive impression (i.e., guessing based on “gut-feeling”). Participants were then exposed to grammatical sequences once a day, for 5 days, during a short-term memory cover task in which the participants were presented with (grammatical) sequences on a computer screen and immediately retyped the sequences on a keyboard without performance feedback. On the last day, participants were again asked to indicate whether they liked or disliked new sequences based to their immediate intuitive impression while event-related fMRI data was acquired.

## Materials and methods

Here, we briefly outline the stimulus material and the experimental procedures used in the current study since these are closely related to those described in Forkstam et al. (2006).

### Participants

Thirty-two healthy right-handed Dutch university students were recruited for the study (50% females, age range: 19–27 years). None of the subjects used any medication, had a history of drug abuse, head trauma, neurological or psychiatric illness, or a family history of neurological or psychiatric illness. All subjects had normal or corrected-to-normal vision. Approval from the local medical ethics committee was obtained and written informed consent was obtained from all participants according to the Declaration of Helsinki.

### Stimulus material

**Figure 1:**
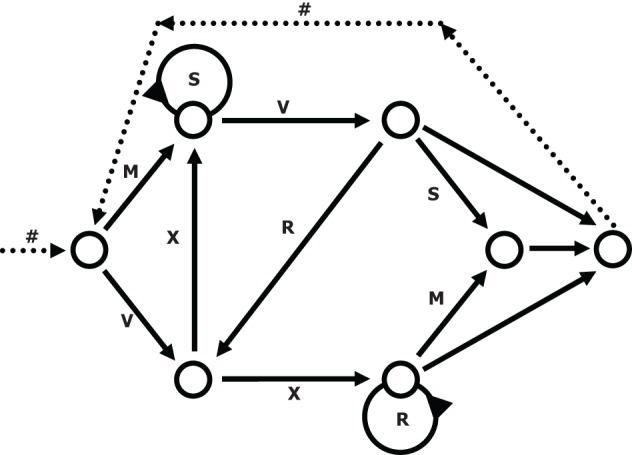
**The transition graph representation of the grammar used in the experiment (cf., Reber, 1967)**.

We used a simple right-linear unification grammar (Figure 1) to generate 569 grammatical (G) sequences, with a sequence length ranging from 5 to 12. For each item we calculated the frequency distribution of 2 and 3 letter chunks for both terminal and complete sequence positions. In this way, we derived a local subsequence familiarity measure termed associative chunk strength (ACS) for each item (Knowlton and Squire, 1996; Meulemans and Van der Linden, 1997; Forkstam et al., 2006, 2008). Local subsequence familiarity, or ACS, is an associative measure that quantifies the superficial resemblance between classification and acquisition sequences. To generate the acquisition set, we randomly selected (in an iterative way) 100 sequences that were representative of the full sequence set in terms of ACS. In the next step, we derived the non-grammatical (NG) sequences from the pool of non-selected G sequences by switching letters in two non-terminal positions. The NG sequences matched the G sequences in terms of terminal and complete sequence ACS. Finally, we randomly selected two sets of 60 sequences each from the remaining G sequences to serve as classification sets. Thus, each classification set consisted of 30 strings of each string type, in other words: 25% high ACS grammatical (HG), 25% low ACS grammatical (LG), 25% high ACS non-grammatical (HNG), and 25% low ACS non-grammatical (LNG). The sequences of high ACS contained subsequences that appeared frequently in the acquisition set, while sequences of low ACS contained subsequences with a low frequency in the acquisition set. See Appendix for a specification and example of the construction of the stimulus material.

### Experimental procedures

The experiment extended over 5 days, including 2 fMRI sessions. On the first day participants had to undergo a preference classification task in the scanner (baseline classification) before any exposure to grammatical sequences during the first acquisition session. On day 2–4, the subjects participated in one behavioral implicit acquisition session each day. On the last (5th) day of the experiment, the subjects underwent a last acquisition session and were then engaged in preference classification during fMRI data acquisition.

### Acquisition

During acquisition, subjects were presented with the 100 acquisition sequences (new randomized order for each acquisition session). Each sequence was centrally presented letter-by-letter on a computer screen (3–7 s corresponding to 5–12 terminal symbols; 300 ms presentation, 300 ms inter-symbol-interval) using Presentation (nbs.neuro-bs.com). The subjects were instructed to retype the sequence on a keyboard. No performance feedback was provided and only grammatical sequences were presented. The acquisition session lasted approximately 20–40 min each day for 5 consecutive days.

### Classification

The classification sequences were organized in a 2 × 2 factorial design with the factors grammaticality status (grammatical/non-grammatical) and local subsequence familiarity (high/low ACS). During the fMRI naive baseline classification on the first day, the participants were presented with letter sequences which they had never seen before (letter-by-letter; 300 ms presentation, 300 ms inter-symbol-interval) and which would not be used during acquisition. They were instructed to indicate, based on their immediate intuitive impression whether they liked or disliked the sequences presented. On the last day of the experiment, subjects underwent an identical preference classification session with novel sequences. The classification sequences were presented via an LCD-projector on semi-transparent screen that the subject comfortably viewed through a mirror mounted on the head-coil. The classification sessions were split in two parts in order to balance response finger within subjects (subjects indicated their classification decision by pushing the corresponding response key with their left/right index finger). After a 1 s pre-stimulus period, the sequences were presented sequentially, letter-by-letter (300 ms presentation, 300 ms inter-symbol-interval), followed by a 3 s response window. A sensorimotor decision baseline task was also included in the fMRI experiment. All conditions, including the sensorimotor decision baseline, were presented in a randomized order during the acquisition of fMRI data both on day 1 and 5. This sensorimotor baseline included sequences of either P or L (e.g., PPPPP or LLLLLLLL), matched to the classification set for sequence length, and presented in the same fashion as the classification sequences. The participants were instructed to respond by pressing the right or left index finger, respectively.

## Data acquisition and analysis

### MR data acquisition

Whole head T2^*^-weighted functional echo planar, blood oxygenation level dependent (EPI-BOLD) fMRI data were acquired with a SIEMENS Avanto 1.5T scanner using an ascending slice acquisition sequence (volume *TR* = 2.6 s, *TE* = 40 ms, 90° flip-angle, 33 axial slices, slice-matrix size = 64 × 64, slice thickness = 3 mm, slice gap = 0.5 mm, FOV = 224 mm, isotropic voxel size = 3.5 × 3.5 × 3.5 mm^3^) in a randomized event related fashion. For the structural MR image volume, a high-resolution T1-weighted magnetization-prepared rapid gradient-echo pulse sequence was used (MP-RAGE; volume *TR* = 2250 ms, *TE* = 3.93 ms, 15° flip-angle, 176 axial slices, slice-matrix size = 256 × 256, slice thickness = 1 mm, field of view = 256 mm, isotropic voxel-size = 1.0 × 1.0 × 1.0 mm^3^).

### fMRI data preprocessing and statistical analysis

We used the SPM software for image preprocessing and statistical analysis (Friston et al., 2007). The EPI-BOLD volumes were realigned to correct for subject movement and corrected for differences in slice acquisition time. The subject-mean EPI-BOLD images were subsequently spatially normalized to the functional EPI template provided by SPM. The normalization transformations were generated from the subject-mean EPI-BOLD volumes and applied to the corresponding functional volumes. The functional EPI-BOLD volumes were transformed into the MNI space, an approximate Talairach space (Talairach and Tournoux, 1988), defined by the SPM template, and spatially filtered with an isotropic 3D spatial Gaussian filter kernel (FWHM = 10 mm). The fMRI data were analyzed statistically, using the general linear model framework and statistical parametric mapping, in a two-step mixed-effects summary-statistics procedure (Friston et al., 2007). We included the realignment parameters for movement artifact correction and a temporal high-pass filter (cycle cut-off at 128 s) to account for various low-frequency effects.

At the first-level, the linear models for the single-subject analyses included explanatory regressors that modeled the sequence presentation period, starting from the violation position in the HNG and LNG conditions and their correct counterparts in the HG and LG conditions. This was done separately for correct and incorrect responses. The initial part of the sequences, before the first critical violation position, was also modeled separately, as was the baseline and the inter-sequence-interval. The explanatory variables were temporally convolved with the canonical hemodynamic response function provided by SPM. At the second-level, we generated single-subject contrast images for the correctly classified HG, LG, HNG, and LNG sequences relative to the sensorimotor decision baseline. These were analyzed in a random-effects repeated-measures ANOVA under an unequal between-conditions variance assumption and with non-sphericity correction for correlated measures. Statistical inference was based on the cluster-size test-statistic from the relevant second-level SPM[T] maps, thresholded at *P* = 0.005 (uncorrected). Only clusters significant at *P* < 0.05 family-wise error (FWE) corrected for multiple dependent comparisons, based on smooth random field theory (Adler, 1981; Adler and Taylor, 2007) are described. In addition, we list the coordinates of local maxima and their corresponding *P*-values corrected for the false discovery rate (Genovese et al., 2002) for descriptive purposes.

## Results

### Classification performance

Some of the behavioral results have been reported in Folia et al. (2008) and are briefly summarized here for convenience. The classification performance (hit rates) on day 1 was at chance level [mean ± standard deviation = 50 ± 7% correct, *T*_(31)_ = 0.42, *P* = 0.67] and increased significantly above chance after 5 days of implicit acquisition [65 ± 14% correct, *T*_(31)_ = 5.7, *P* < 0.001]. Standard signal detection analysis confirmed a robust d-prime effect in discriminating between grammatical (G) and non-grammatical (NG) sequences one day 5 (d-prime: 0.94) but not on day 1 [d-prime: 0.006; day 5 vs. day 1: *T*_(31)_ = 4.91, *P* < 0.001]. No significant response bias was found (beta-value: day 1 = 1.02; day 5 = 1.02; all *P* > 0.6). Moreover, participants did not discriminate between high and low ACS sequences (d-prime: day 1 = 0.15; day 5 = 0.22; all *P* > 0.66). This suggests that there is no difference in the ability to discriminate sequences based on local subsequence familiarity. No significant response bias was found (beta-value: day 1 = 1.02; day 5 = 1.01; all *P* > 0.6).

**Figure 2:**
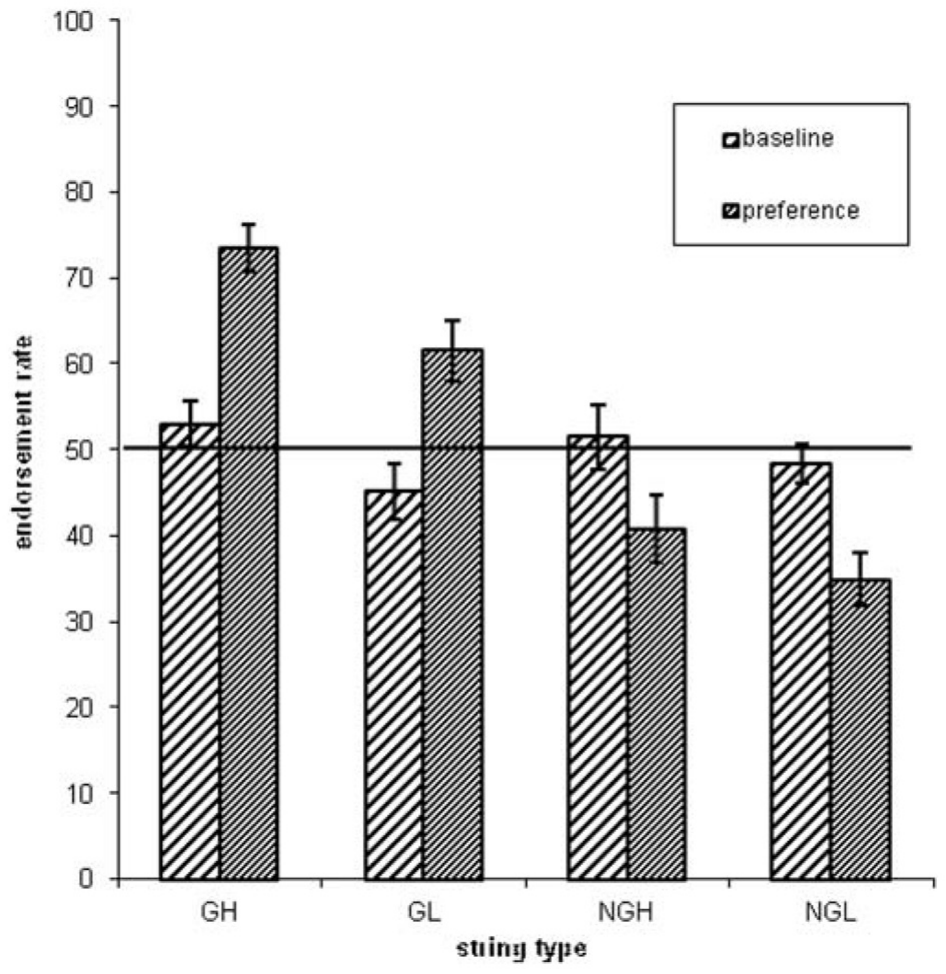
**The endorsement rates as a function of grammaticality status (G = grammatical sequences, NG = non-grammatical sequences) and associative chunk strength (H = high ACS sequences, L = low ACS sequences)**. Error bars correspond to standard error of the mean.

**Figure 3:**
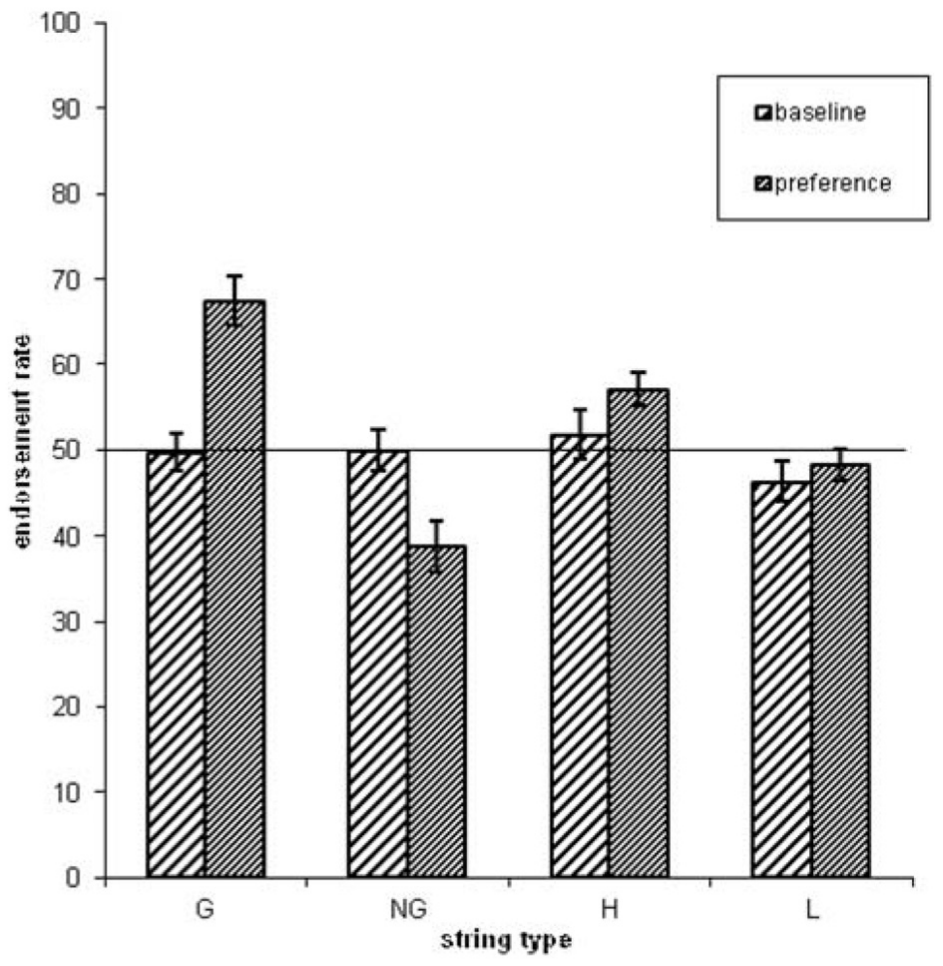
**The endorsement rates as a function of grammaticality status and associative chunk strength (G = grammatical, NG = non-grammatical, H = high ACS, L = low ACS sequences)**. Error bars correspond to standard error of the mean.

Concerning the endorsement rate (i.e., items preferred independent of actual grammaticality status), a repeated-measures ANOVA showed that both grammaticality status and ACS influenced preference classification on day 5, but not on day 1 [grammaticality day 1: *F*_(1, 31)_ = 0.00, *P* = 0.95, η^2^_*p*_ = 0.00; ACS day 1: *F*_(1, 31)_ = 2.3, *P* = 0.14, η^2^_*p*_ = 0.06; interaction grammaticality and ACS on day 1: *F*_(1, 31)_ = 2.3, *P* = 0.13, η^2^_*p*_ = 0.07; Table 1, Figures 2, 3]. There was no effects of grammaticality status for either high or low ACS sequences on day 1 [HG vs. HNG: *F*_(1, 31)_ = 0.42, *P* = 0.52, η^2^_*p*_ = 0.01; LG vs. LNG: *F*_(1, 31)_ = 0.65, *P* = 0.43, η^2^_*p*_ = 0.02]. In contrast, on day 5, the endorsement rate was significantly affected by the grammaticality status [*F*_(1, 31)_ = 31.7, *P* < 0.001, η^2^_*p*_ = 0.50], local subsequence familiarity [*F*_(1, 31)_ = 15.4, *P* < 0.001, η^2^_*p*_ = 0.33], while the interaction between grammaticality and ACS was non-significant [*F*_(1, 31)_ = 3.8, *P* > 0.05, η^2^_*p*_ = 0.11]; this was also the case for sequences with high and low subsequence familiarity, respectively, [HG vs. HNG: *F*_(1, 31)_ = 34, *P* < 0.001, η^2^_*p*_ = 0.52; LG vs. LNG: *F*_(1, 31)_ = 24, *P* < 0.001, η^2^_*p*_ = 0.44]. During each classification session (day 1/5) the subjects were asked to rate their level of attention (VAS ratings, four times evenly distributed over each session). There was no significant attention difference between days (day 1: 7.9, *SD* = 1.07; day 5: 7.9, *SD* = 1.12).

**Table 1:** **Endorsement rates over grammaticality and local subsequence familiarity (ACS) for day 1 and day 5**.

	**Day 1**		**Day 5**	
	**High ACS (%)**	**Low ACS (%)**	**High ACS (%)**	**Low ACS (%)**
G	53 (15)	45 (18)	73 (16)	62 (20)
NG	51 (21)	48 (13)	41 (22)	34 (17)

Percentage of items endorsed by condition; mean performance level and standard deviation (SD). G, grammatical; NG, non-grammatical; ACS, associative chunk strength.

**Table 2:** **Overlap between the activated clusters in the listed studies and the clusters that we found activated in the left inferior frontal region related to the learning effect (Figure 7)**.

**Study**	**[*x, y, z*]**	**Function**	**Cluster**	**Nearest**	***P***
Friederici et al., 2006	[−36, 20, −2]	nested	*P*_FWE_ = 0.004	[−36, 20, −2]	<0.001
	[−46, 16, 8]	nested	*P*_FWE_ = 0.001	[−48, 18, 6]	0.003
Opitz and Friederici, 2007	[−47, 12, 24]	non-adjacent	*P*_FWE_ = 0.001	[−48, 12, 24]	0.001
Bahlmann et al., 2008	[−46, 5, 16]	nested	*P*_FWE_ = 0.016	[−50, 10, 18]	0.003
	[−34, 28, 22]	nested	*P*_FWE_ = 0.021	[−38, 24, 22]	0.004

Columns 1–3: The [x, y, z] coordinates and the function labeling are taken from the AGL studies of Friederici et al. (2006), Opitz and Friederici (2007), and Bahlmann et al. (2008). Opitz and Friederici (2007) actually report an effect of non-adjacent dependency processing in the opercular part of the left inferior gyrus [−47, −12, 24]. However, with a y = −12, this is localized in or posterior to the central sulcus, so we interpret the y-coordinate as y = 12. Here we do a local search in a spherical region, centered on the coordinates provided, with a radius of 13 mm. This radius correspond to the estimated spatial standard deviation (localization precision) from the syntax related data provided by Bookheimer (2002; recently replicated by Hagoort and Indefrey, 2014) and quantified in Petersson et al. (2004). Columns 4–6: the cluster P-values, the nearest supra-threshold voxel with corresponding P-values are from the current study (Friston, 1997; Worsley, 2003).

### fMRI results

**Figure 4:**
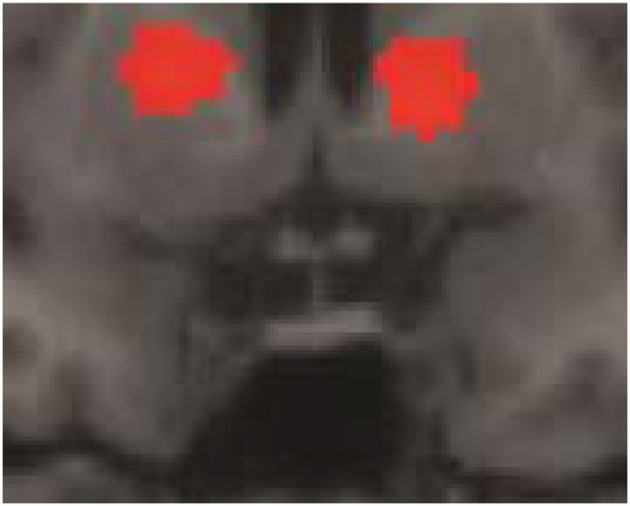
**Basal ganglia activations**. Preference classification vs. sensorimotor decision baseline on day 5. The effect was present but smaller on day 1 (day 5 vs. day 1; cluster *P*_FWE_ = 0.012; [x, y, z] = [−24, −6, −4], *P*_FDR_ = 0.012, small-volume correction).

**Figure 5:**
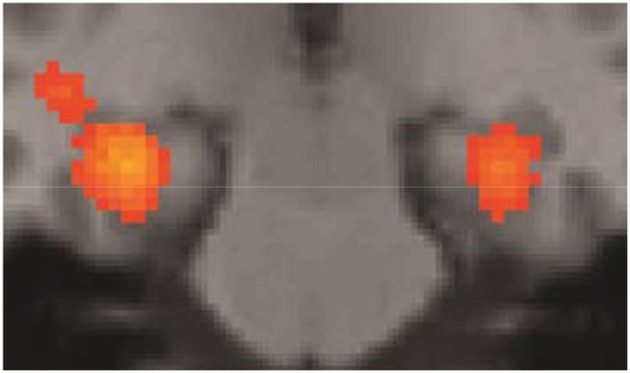
**Medial temporal lobe deactivations**. Sensorimotor decision baseline vs. preference classification on day 5 (all clusters *P*_FWE_ < 0.001). These effects were very similar on day 1.

Some of the fMRI results were summarily described in Folia et al. (2011), in particular, the overlap between grammaticality- and preference classification on day 5 was tested and reported. Here we report the fMRI results from the complete learning design experiment described in the current study. When compared to the sensorimotor decision baseline, preference classification activated a set of regions (*P*_FWE_ < 0.001) previously found to be involved in grammaticality classification (Petersson et al., 2004, 2010; Forkstam et al., 2006), including the inferior and middle frontal regions bilaterally and the anterior cingulate cortex (all clusters *P*_FWE_ < 0.001). Bilateral posterior activations included the inferior parietal, the posterior cingulate, and the occipital cortex. Moreover, the basal ganglia (caudate/putamen/globus pallidus) were activated during classification (relative the sensorimotor decision baseline; Figure 4) and this increased over the 5 days of implicit acquisition (cluster *P*_FWE_ = 0.012; [*x*, y, z] = [−24, −6, −4], *P*_FDR_ = 0.012, small-volume correction). In contrast, the medial temporal lobes were deactivated (right cluster *P*_FWE_ < 0.001; [30, −24, −16], *P*_FDR_ = 0.007; left cluster *P*_FWE_ < 0.001; [−30, −28, −12], *P*_FDR_ = 0.001; Figure 5). These effects extended along most of the medial temporal lobe axis, bilaterally.

**Figure 6:**
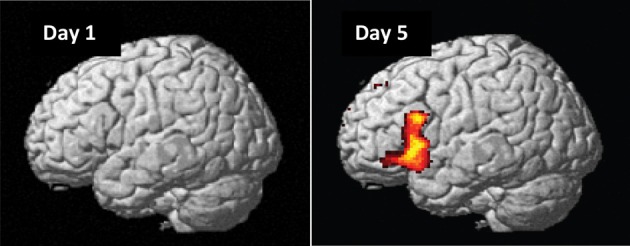
**Grammaticality effect during preference classification**. Brain regions engaged by artificial syntactic anomalies (NG > G). Day 1: No significant effect in the left hemisphere (*P*_FWE_ > 0.50). Day 5: Significant activation in the inferior frontal (left and right: *P*_FWE_ < 0.001) and medial prefrontal (*P*_FWE_ < 0.001; not shown) regions.

On day 1, as expected, we found no significant main effects or interactions for naïve preference classification, except an initial bias activations in the right superior-inferior parietal region (BA 7/40; G > NG, *P*_FWE_ = 0.002) and an interaction in the right posterior cingulate cortex (BA 23/31; [HNG—HG] > [LNG—LG], *P*_FWE_ = 0.001). Importantly, these initial bias effects reversed and disappeared with repeated implicit exposure to grammatical sequences. After 5 days, preference classification resulted in several significant brain activations (Figure 6, Table 3).

**Table 3:** **Preference classification Day 5**.

**Anatomical region**	**Brodmann's area**	**[*x, y, z*]**	***Z* score**	***P*-value**
***Left inferior frontal cluster***				**<*0.001***
L inferior frontal gyrus	BA 44	−54, 14, 2	4.07	0.013
	BA 44/45	−60, 20, 16	3.90	0.016
	BA 44/45	−52, 18, 22	3.81	0.019
	BA 45	−60, 22, 10	3.76	0.020
	BA 45/47	−56, 18, 2	4.02	0.014
	BA 47	−42, 20, −10	3.90	0.016
L mid-anterior insula	BA 13/15	−38, 14, −10	4.06	0.013
L frontal operculum/anterior insula	BA 49/15	−38, 22, −4	3.51	0.030
***Right inferior-middle frontal cluster***				**<*0.001***
R inferior frontal gyrus	BA 44/45	50, 24, 18	3.65	0.023
	BA 45	56, 30, 12	3.54	0.028
	BA 47	46, 32, −4	5.08	0.010
R mid-anterior insula	BA 13/15	40, 20, −6	4.15	0.011
R frontal operculum/anterior insula	BA 49/15	36, 20, −10	3.87	0.017
R inferior-middle frontal gyrus	BA 45/46	46, 34, 12	3.41	0.036
	BA 46	52, 40, 18	3.27	0.044
	BA 8/9	50, 20, 44	3.45	0.033
***Medial prefrontal cluster***				**<*0.001***
	BA 8	0, 26, 52	4.41	0.010
	BA 8/32	6, 30, 44	4.60	0.010
	BA 32	−4, 32, 28	3.16	0.053
	BA 24/32	10, 32, 24	4.27	0.010
	BA 10	16, 60, 22	4.45	0.010
	BA 9/10	12, 52, 24	3.52	0.029

Significant effects observed for non-grammatical vs. grammatical sequences (grammaticality effect). Cluster P-values are family-wise error corrected and P-values of local maxima are corrected for false-discovery rate.

In particular, artificial syntactic anomalies (grammaticality effect, NG > G) engaged the left inferior and right inferior-middle frontal gyri (left and right cluster *P*_FWE_ < 0.001) centered on Broca's region (BA 44/45), extending into BA 47 and the right middle frontal gyrus (BA 46) as well as the frontal operculum/anterior insula. Additional activations were found in the medial prefrontal regions (BA 8/32; cluster *P*_FWE_ < 0.001), while no significant activations were observed in the reverse contrast (G > NG; cluster *P*_FWE_ > 0.54). We found no significant effect of local subsequence familiarity (all clusters *P*_FWE_ > 0.98) and no significant interactions (all clusters *P*_FWE_ > 0.83), consistent with our previous behavioral findings.

**Figure 7:**
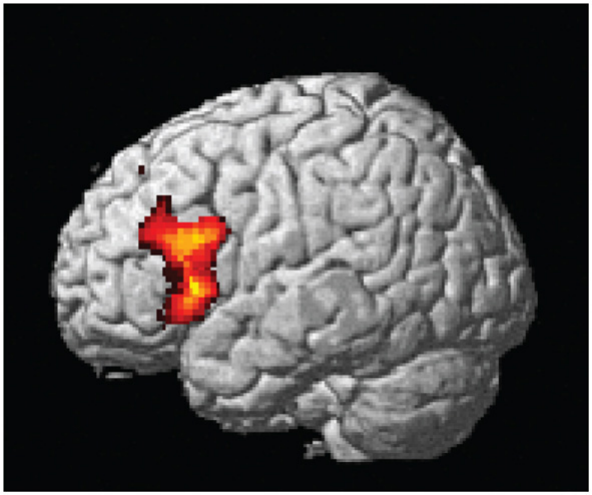
**Learning effect with respect to grammaticality status**. Comparing the grammaticality effect (NG > G) during preference classification on day 5 and day 1 yielded significant effects in the inferior frontal (left and right: *P*_FWE_ < 0.001), the medial prefrontal (*P*_FWE_ < 0.001; not shown) regions.

The central result of this study is that all the artificial syntax processing effects observed on day 5 resulted from the exposure to grammatical items generated from the underlying grammar during the 5 days of implicit acquisition (Figure 7, Table 4). In particular, for the day 5 vs. day 1 comparison of the NG vs. G effect, we found the same set of brain regions that was observed on day 5, including the inferior frontal (BA 44/45; cluster *P*_FWE_ < 0.001) and medial prefrontal region (BA 8/32; cluster *P*_FWE_ < 0.001; Figure 7, Table 4). In addition, we confirmed that the initial bias activation observed in the right superior-inferior parietal region on day 1 had disappeared (BA 7/40; cluster *P*_FWE_ = 0.021). No other learning effects reached significance (all clusters *P*_FWE_ > 0.90).

**Table 4:** **Implicit learning effects**.

**Anatomical region**	**Brodmann's area**	**[*x, y, z*]**	***Z* score**	***P*-value**
***Left inferior frontal cluster***				**<*0.001***
L inferior frontal gyrus	BA 44	−60, 18, 6	3.58	0.048
	BA 44/45	−60, 22, 10	3.84	0.035
	BA 45	−56, 16, 0	3.72	0.040
	BA 45/47	−38, 22, 0	3.80	0.037
***Right inferior-middle frontal cluster***				**<*0.001***
R inferior frontal gyrus	BA 44/45	58, 20, 6	4.01	0.030
	BA 45	52, 28, 16	4.11	0.027
	BA 45/47	38, 24, −4	3.78	0.038
R inferior-middle frontal gyrus	BA 45/46	48, 32, 16	3.99	0.031
	BA 46	48, 40, 18	3.68	0.042
***Inferior parietal cluster***				***0.021***
R inferior parietal cortex	BA 40	44, −42, 44	3.90	0.034
	BA 40	44, −46, 40	3.74	0.039
	BA 7/40	44, −46, 48	3.68	0.042
***Medial prefrontal cluster***				**<*0.001***
	BA 8/32	4, 32, 48	4.96	0.018
	BA 8/32	0, 22, 52	4.47	0.020
	BA 8/32	−2, 22, 48	4.46	0.020
	BA 32	10, 30, 32	3.58	0.048

Significant learning effects in relation to grammaticality status (non-grammatical vs. grammatical sequences) on day 5 compared to on day 1. Cluster P-values are family-wise error corrected and P-values of local maxima are corrected for false-discovery rate.

## Discussion

In the present event-related fMRI study we investigated the effect of 5 days of implicit acquisition on preference classification by means of an AGL paradigm based on the structural mere-exposure effect using a simple right-linear unification grammar. This is the first fMRI study to investigate implicit AGL with preference classification in a proper learning design (i.e., including baseline measurements prior to grammar exposure). The main fMRI findings are consistent with previous grammaticality classification results (Petersson et al., 2004, 2010; Forkstam et al., 2006; Folia et al., 2011). Importantly, and central to this study, we show that the preference classification results are the outcome of the underlying implicit learning process. More specifically, after 5 days of implicit acquisition, the fMRI results showed activations in a network of brain regions including the inferior frontal regions (centered on BA 44/45) and the medial prefrontal region (centered on BA 8/32; Figure 6, Table 3). The inclusion of a naive preference classification fMRI baseline measurement in a learning design (Petersson et al., 1999a,b) allow us to conclude that the fMRI findings are the intrinsic outcomes of the learning process itself and not a reflection of a preexisting functionality that is recruited during classification, independent of acquisition (Figure 7, Table 4). Moreover, the presence of initial bias activations observed during the naive preference classification (e.g., right superior inferior parietal region) emphasizes the importance of including fMRI baseline measurements in learning designs. Similar initial bias effects are sometimes observed in behavioral data (Forkstam et al., 2008), although not in the present data, which can thus, be less sensitive in this respect compared to fMRI. Behavioral results (Forkstam et al., 2008) suggest that the presence of right hemisphere activation on the first day, during naive classification, might be related to an initial attempt by the participants to subjectively classify the sequences based on spurious surface features attributed to a prior participant bias. Importantly, these bias activations reversed with repeated exposure to grammatical sequences and were not observed on the last day of the experiment. Nevertheless, this emphasizes the importance of including naive fMRI baseline measurements in order to properly characterize the learning related effects (Petersson et al., 1999a,b).

At the behavioral level, participants incidentally learned relevant aspects associated with the underlying grammar and were able to successfully generalize to new sequences after 5 days of implicit acquisition. In contrast, the classification performance was at chance-level for the naive preference classification. The learning effect with respect to superficial local subsequence familiarity, although significant, was smaller (ACS: η^2^_*p*_ = 0.33; compared to grammaticality status: η^2^_*p*_ = 0.50). This finding was more pronounced in the fMRI results, which showed no significant learning effect of ACS. In contrast, the effect of grammaticality status, resulting from 5 days of implicit acquisition, was highly significant. Additional support for the implicit nature of the knowledge utilized during preference classification on day 5 come from the fact that the basal ganglia (Figure 4) were activated during classification. This is perhaps not surprising, given the massive nature of the recurrent connectivity between the frontal neocortex and the basal ganglia (i.e., fronto-striatal loops). It is hard to imagine fully functioning prefrontal regions without normally functioning basal ganglia and there is evidence that the basal ganglia are involved in rule-processing (e.g., Packard and Knowlton, 2002; Ullman, 2004; Forkstam and Petersson, 2005; Teichmann et al., 2005, 2008). In contrast, the medial temporal lobe memory system was consistently deactivated in this study. The medial temporal lobes are associated with explicit declarative memory (Squire, 1992; cf., Petersson et al., 1997, 1999a), while the basal ganglia have been related to implicit learning and the procedural memory system (Seger, 1994; Packard and Knowlton, 2002; Ullman, 2004; Forkstam and Petersson, 2005). However, the implicit procedural memory system (related to the basal ganglia) and the explicit declarative memory system (related to the medial temporal lobes) are not necessarily always engaged in opposition. The experimental evidence suggests a more complex picture where these two memory systems can interact both in a competitive and a cooperative, non-competitive manner (Devan and White, 1999; Voermans et al., 2004; Brown et al., 2012). However, the interpretation of this state of affairs is not well-understood, except perhaps, to suggest that several neural learning mechanisms can be recruited depending on the type of information processing the brain engages in, in any particular context. The results of the present study, as well as grammaticality classification fMRI studies based on implicit AGL (Forkstam et al., 2006; Petersson et al., 2010), show strong activation and deactivation of the basal ganglia and the medial temporal lobes, respectively.

The sequential presentation mode used in this study entails on-line processing memory (i.e., something roughly akin to a “working memory”). We often use sequential instead of whole sequence presentation in order to model the sequential nature of language input/output. This aspect of the experimental paradigm is very unlikely to affect the reported results or their interpretation. First, the demand for on-line processing memory in the preference classification task is the same for all sequence types (matched for length; and grammatical/non-grammatical sequences matched for ACS). In the case of grammaticality classification, it might be the case that there is a tendency that the non-grammatical sequences require somewhat less processing memory compared to grammatical sequences, since the participants could in principle stop processing the non-grammatical items as soon as they judge them non-grammatical. However, this would not explain the observed frontal activation increases observed for non-grammatical compared to grammatical items during grammaticality classification (Petersson et al., 2004, 2010; Forkstam et al., 2006). Second, Petersson et al. (2004) used whole sequence presentation in a grammaticality classification task and reported virtually identical results as found in the present study after 5 days of implicit acquisition. Therefore, the presentation mode of the stimulus items (whole/sequential) seems to be of little consequence for the fMRI results.

### The inferior frontal region, AGL, and other cognitive domains

Human languages are characterized by “design features” (Hockett, 1963, 1987; including discreteness, arbitrariness, productivity, and the duality of patterning) and somehow these characteristics arise from the properties of the human brain, how it develops and learns in interaction with its environment. One of the difficulties with acquiring a language is related to the fact that the internal mental structures that represent linguistic information are not expressed in the surface form of the language (i.e., the utterance). This suggests that humans are equipped with learning mechanisms which shape the acquired language into a discrete and recursively organized system when the relevant communicative context is present. With respect to syntax, these learning mechanisms are to a large extent implicit in nature and despite much progress it is still not well-understood how humans acquire their native language skills (Folia et al., 2010, 2011; Reber, 2011).

AGL was originally implemented in order to investigate implicit learning mechanisms shared with natural language acquisition (Reber, 1967). The neurobiology of implicit sequence learning, assessed with AGL, has been investigated by means of functional neuroimaging (Petersson et al., 2004; Forkstam et al., 2006), brain stimulation (Udden et al., 2008; de Vries et al., 2010), and has consistently shown that Broca's region (BA 44/45), in addition to other brain regions, is involved. In addition, the breakdown of syntax processing in agrammatic aphasia and in patients with lesions in the inferior frontal region is associated with impairments in AGL (Christiansen et al., 2010; Opitz and Kotz, 2012) and individual variability in implicit sequence learning correlates with language processing (Conway and Pisoni, 2008; Misyak et al., 2010). Taken together, this supports the idea that AGL taps into implicit learning/processes that are shared with aspects of natural syntax acquisition and processing.

In this study we used an implicit AGL paradigm, based on preference classification and the structural mere-exposure effect. One difference between this type of paradigm and explicit AGL paradigms that have been used lately is that in the former, both the acquisition and classification phases are implicit and no reference to any previous acquisition episode made. Because of this, it is never necessary to inform the participants about the existence of an underlying grammar or any other aspect of the paradigm and from the subject's perspective there is no correct or incorrect response. In several functional neuroimaging studies, explicit paradigms have been used (e.g., Friederici et al., 2006; Opitz and Friederici, 2007; Bahlmann et al., 2008, 2009; reviewed in Petersson et al., 2010). For example, in the studies by Opitz and Friederici (2003, 2007); Opitz and Kotz (2012), the participants were instructed to extract the underlying rules during training, while feedback was provided on each trial during testing. Moreover, while the artificial language used by Opitz and Friederici (2003) is finite (Figure 1, p. 1731), in the modified version (Opitz and Friederici, 2007; Opitz and Kotz, 2012), they introduce a “complementizer” in a way that yields a right-branching regular language (Figure 1, p. 586, Opitz and Friederici, 2007; note also that both conditions depicted correspond to hierarchical phrase structures). It is worth noting in this context, that regular grammars can generate non-adjacent (long-distance) dependencies (cf., e.g., Pullum and Scholz, 2010; see also Pullum and Scholz, 2009, and in particular the supporting on-line material of Petersson and Hagoort, 2012, for simple examples). We emphasize that the use of a particular grammar in AGL does not ensure that the participants acquire, or use, this during testing, instead of using, for example, a different and perhaps simpler way of representing the knowledge acquired (de Vries et al., 2011, 2012). Finally, it should be noted that the representational structures that function during explicit decision-making are not the same as those that hold the knowledge of the structure that is used to make those decisions. Here, we have used the notions “implicit” and “implicit learning” in their classical sense, which entails a lack of meta-cognitive knowledge/judgment and in particular the absence of any stated use of explicit “problem solving” strategies. For example, when we speak we are clearly aware of the fact that we produce sentences, but we have no explicit knowledge or insight into how this is actually carried out.

It is unlikely that explicit selection or any other form of explicit decision making can explain our findings in any relevant sense for another reason. In the preference classification task, there is as much “decision making” going on whether the participant likes or dislikes an item. Moreover, the sensorimotor baseline of this study included an explicit decision component and the fact that we find the same inferior frontal activations centered on Broca's region (BA 44/45) in both preference and grammaticality classification, suggests to us, that the observed activation reflects neural processing related to implicit knowledge. We note that the unification grammar framework offers an alternative perspective on selection and control in this context. In this picture, it is the syntactic features of lexical items that exert control over the integration process via a general integration mechanism, which is already in place, for unifying structured representations (cf., Vosse and Kempen, 2000; Jackendoff, 2002; Petersson et al., 2005). Thus, control is implicitly distributed over a long-term memory representation, the mental lexicon, in terms of the control features that govern the integration process based on what is allowed (or not) to merge.

It is uncontroversial that participants have acquired some relevant knowledge associated with the underlying grammar, if they, for example, discriminate new grammatical from non-grammatical items in a reliable manner. However, this does not necessarily imply that the participants process the sequences according to the rules of the grammar and the empirical findings rarely support such claims in any strong sense (cf., Petersson et al., 2010; Petersson and Hagoort, 2012, for a discussion).

For example, sometimes it appears as if claims are made that different subregions of Broca's region are specifically related to different types of grammars or the processing of, for example, nested non-adjacent dependencies. In this study we used a simple right-linear unification grammar and in Table 2 we specify the overlap between the learning effects observed in the left inferior frontal region in this study (Figure 7) and the activated clusters reported in some of the studies previously reviewed. The outcome of this comparison suggests that the left inferior frontal region (BA 44/45) is significantly related to implicit AGL and artificial syntax processing, independent of the fact that the simple right-linear unification grammar we investigated does not involve nested center-embedded non-adjacent dependencies or dependencies introduced by syntactic displacement (i.e., syntactic movement). These findings are similar to corresponding findings reported in Petersson et al. (2010) for the grammaticality instruction. Thus, in the context of artificial syntax processing, and more generally language processing, the left inferior frontal region is unlikely to be specific to the processing of or nested center-embedded structures or non-adjacent dependencies introduced by syntactic movement. Instead, these results, in conjunction with previous functional neuroimaging results, suggest that the left inferior frontal region is a generic on-line structured sequence processor that unifies information from various sources in an incremental and recursive manner (for a discussion see Petersson et al., 2010; Petersson and Hagoort, 2012).

Several previous studies have suggested that the left inferior frontal region has a broader role in cognition than just language processing (Marcus et al., 2003; Petersson et al., 2004; Hagoort, 2005), including action recognition and movement preparation (e.g., Thoenissen et al., 2002; Hamzei et al., 2003), musical syntax (e.g., Maess et al., 2001; Koelsch et al., 2002; for a review see Patel, 2003), lexical and sub-lexical processing (Sahin et al., 2009), working memory (Price, 2010), and visuo-spatial sequence processing (Bahlmann et al., 2009). Thus, a growing body of evidence from functional neuroimaging suggests that the processing of structural sequence relations in several cognitive domains overlap in the inferior frontal regions, including language, music and artificial grammars/languages. This suggests a framework for the left inferior frontal region in which incremental recursive (i.e., state-dependent) integration of various sources of linguistic information (e.g., phonological, syntactic, semantic/pragmatic) operate interactively in parallel via interfaces (cf., e.g., Jackendoff, 2007). Moreover, other brain regions have been related to the processing of natural language syntax, including the left inferior parietal region, the left superior and middle temporal regions as well as right hemisphere, largely homotopic, regions (e.g., Snijders et al., 2009, 2010; Segaert et al., 2012; for reviews see Bookheimer, 2002; Price, 2010; Friederici, 2012; Hagoort and Indefrey, 2014). Finally, none of these regions seem uniquely related to syntax processing (Petersson et al., 2004; Petersson and Hagoort, 2012). It is therefore not unreasonable to suggest that artificial and natural syntax processing, and more generally language processing, is dependent on a functional network of interacting brain regions (Friederici, 2012; Petersson and Hagoort, 2012), none perhaps which is uniquely involved in syntax processing only. This conclusion appears to hold for higher cognitive functions more generally (Ingvar and Petersson, 2000; Petersson et al., 2009).

### Acquisition of structured sequence knowledge

The acquisition of language is a complex learning task which is governed by constraints derived from the properties of the developing human brain. The current lack of knowledge concerning the actual mechanisms involved during infancy makes it difficult to determine the relative contributions of innate- and acquired knowledge in language acquisition (Folia et al., 2010, 2011; Petersson and Hagoort, 2012). On the traditional Chomskyan view, disputed by many (for a recent example, see Reber, 2011; for a discussion see Petersson and Hagoort, 2012), the input underdetermines the linguistic knowledge of the adult language capacity. Thus, the acquisition of a grammar is not only based on an analysis of the linguistic input, but depends on an innate structure (i.e., the “language acquisition device”) that guides the acquisition process (Jackendoff, 2002, 2007). In this context, it is of interest to note that Folia et al. (2011) reported behavioral and corresponding activation differences in Broca's region (BA 44/45), in an implicit AGL grammaticality classification paradigm, which depended on the genotype related to the CNTNAP2 gene, a gene controlled by the transcription factor FOXP2.

In the following, we briefly discuss work on the acquisition of structured sequence knowledge (for reviews see Gomez and Gerken, 2000; Folia et al., 2010), which seem relevant to the current study. Uddén et al. (2009, 2012) investigated implicit acquisition of nested- and crossed non-adjacent dependencies (corresponding to context-free and context-sensitive grammars, respectively), while controlling for local subsequence familiarity, in an implicit learning paradigm based on structural mere-exposure in a paradigm very similar to the current study. Given the difficulty reported by some researchers in getting participants to acquire non-adjacent dependencies, the repeated exposure to grammatical items over 9 days used by Uddén et al. (2009, 2012) was likely important. In particular, this provides exposure and presumably time for both the necessary abstraction and knowledge consolidation processes to take place. There is some experimental evidence suggesting that this is important for improved performance in implicit AGL. For example, sleep has been shown to have a significant effect on grammaticality classification after implicit AGL (Nieuwenhuis et al., 2013), and to promote abstraction processes after AGL in infants (Gomez et al., 2006). Uddén et al. (2009, 2012) found that, while the subjects implicitly acquired knowledge about the non-regular nested structures, the acquisition of non-regular dependencies were harder compared to regular dependencies in the underlying grammar. Participants in these studies also acquired sensitivity to a context-sensitive agreement structure that generated non-adjacent crossed dependencies, but found the agreement violations harder to reject than category violations (Uddén et al., 2009, 2012). Interestingly, in an ERP study by Friederici et al. (2011), they reported that 4-months-old infants developed sensitivity to a simple non-adjacent AXB-dependency structure, perhaps suggesting that the negative results in 12-months-old reported by Gomez and Maye (2005) might be due to a lack of sensitivity. The ability to develop sensitivity to both adjacent and non-adjacent dependencies from early infancy suggests that innate implicit learning mechanism(s) are present already in the new born. Friederici et al. (2011) reported that the grammaticality effect (NG vs. G) yielded a late centro-parietal positivity and in a parallel experiment on adults, the same paradigm yielded a P600 (Mueller et al., 2009), which often reflects processes related to syntax (Hagoort et al., 1993).

## Conclusion

We conclude that preference classification, in combination with a structural mere-exposure design, can be used to investigate structural (syntax) processing in unsupervised AGL paradigms with event-related fMRI in proper learning designs. The main findings suggest that a network of brain regions, including the inferior frontal (centered on BA 44/45) and the medial prefrontal regions (centered on BA 8/32), are activated as the intrinsic result of an implicit learning process. Support for the implicit nature of the knowledge utilized during preference classification come from the fact that the basal ganglia were activated during classification, while the medial temporal lobe memory system was consistently deactivated.

### Conflict of interest statement

The authors declare that the research was conducted in the absence of any commercial or financial relationships that could be construed as a potential conflict of interest.

## Appendix

**Table:**

***HG items***	**cACS**	**tACS**	***HNG items***	**cACS**	**tACS**
MSSVRXVRXVS	61.47	22.75	M**X**SVRXVRX**S**S	61.79	23.25
MSVRXVRXRRRR	53.38	23.00	MSVRXVRX**SX**RR	53.33	25.25
MVRXSSSVRXR	63.63	27.00	MVRX**RX**SVRXR	61.32	27.00
MSVRXSSSSSSV	58.52	49.75	MSVRXSS**VR**SSV	58.76	49.75
VXVRXSSSSSSV	55.48	51.50	VXVRXSS**VR**SSV	55.71	51.50
***LG items***	**cACS**	**tACS**	***LNG items***	**cACS**	**tACS**
VXSVRXRRRM	46.76	22.00	VXSVRX**VX**RM	46.82	20.75
MSSSVRXRRRM	48.05	23.00	MSSSVRX**VX**RM	48.11	21.75
MSSSSSSSVS	44.76	22.75	MSS**VR**SSSVS	45.06	22.75
VXSSSSSSSVS	44.95	21.75	VXSS**VR**SSSVS	45.21	21.75
MSSSSSSSSVS	44.95	22.75	MSS**VR**SSSSVS	45.21	22.75

Example of the stimulus material used in the present experiment. HG, high grammatical; HNG, high non-grammatical; LG, low grammatical; LNG, low non-grammatical; cACS, frequency distribution of 2 and 3 letter chunks for complete sequence position (in relation to the acquisition stimuli); tACS, frequency distribution of 2 and 3 letter chunks for terminal sequence position. The non-grammatical (NG) items were derived from the grammatical (G) sequences, by switching letters in two non-terminal positions (in bold). The NG sequences matched the G sequences in terms of terminal and complete sequence ACS. This was accomplished by generating all possible NG sequences for each G sequence, and selecting the NG sequence that was most equal in ACS to the G sequence. Each letter sequence is decomposed into 2 and 3 letter chunks, their frequency for complete and terminal position in the learning sequences are calculated. Example of the calculation of the complete sequence position (cACS): MSSVRXVRXVS is decomposed in the bigrams MS (40), SS (59), SV (87), VR (97), RX (97), XV (50), VR (97), RX (97), XV (50), VS (16). The frequencies in the learning sequences of these bigrams are shown in parenthesis. The sequence was also decomposed in the trigrams, MSS (27), SSV (59), SVR (75), VRX (97), RXV (37), XVR (41), VRX (97), RXV (37), XVS (8). The cACS of this item was calculated by averaging its different bigram and trigram frequencies. The obtained cACS is 61.47 indicates a relatively high mean high frequency of 2 and 3 letter chunks in relation to the acquisition stimuli.

**Table A1:** **Characteristics of the stimulus material used in the present experiment**.

	***n***	**Mean ACS**	**% of stimulus type per sequence length**			
			**5–6**	**7–8**	**9–10**	**11–12**
**ACQUISITION SET**						
	100	59.1 (7.8) [37.6–72.6]	2	16	30	52
**CLASSIFICATION SET**						
GH	30	60.0 (5.2) [50.1–71.3]	0	10	20	70
GL	30	40.8 (8.6) [20.5–48.8]	10	10	37	43
NGH	30	59.2 (5.4) [49.2–77.0]	0	10	20	70
NGL	30	40.9 (8.6) [20.5–49.8]	10	10	37	43

ACS, associative chunk strength; G, grammatical; NG, non-grammatical, H, high ACS; L, low ACS. Values in parenthesis are standard deviations, values in brackets indicate ranges. Percentage of stimulus type per sequence length indicates the percentage of sequences in the acquisition and classification set ranging from 5 to 12 letters (length).
